# Self-Lubricanting Slippery Surface with Wettability Gradients for Anti-Sticking of Electrosurgical Scalpel

**DOI:** 10.3390/mi9110591

**Published:** 2018-11-13

**Authors:** Guang Liu, Pengfei Zhang, Yang Liu, Deyuan Zhang, Huawei Chen

**Affiliations:** 1 School of Mechanical Engineering and Automation, Beihang University, Beijing 100191, China; liuguang0701@buaa.edu.cn (G.L.); liuyang168@buaa.edu.cn (Y.L.); zhangdy@buaa.edu.cn (D.Z.); 2 Beijing Advanced Innovation Center for Biomedical Engineering, Beihang University, Beijing 100191, China; pengfei89.zhang@gmail.com; 3 Department of Bioengineering and Therapeutic Sciences, University of California, San Francisco, CA 94158, USA

**Keywords:** self-lubricating slippery surface, wettability gradient, electrosurgical scalpels, anti-sticking, soft tissue

## Abstract

Soft tissue sticking on electrosurgical scalpels in minimally invasive surgery can increase the difficulty of operation and easily lead to medical malpractice. It is significant to develop new methods for anti-sticking of soft tissue on electrosurgical scalpels. Based on the characteristics of biomimetic ultra-slippery surface, a self-lubricating slippery surface with wettability gradients on electrosurgical scalpel was designed and fabricated. Non-uniformly distributed cylindrical micro pillars, which constitute the wettability gradients, were prepared by an electrolytic etching process and the theoretic of the spontaneous liquid spreading process was analyzed. The silicophilic property of wettability gradients surface was modified by octadecyltrichlorosilane (OTS) self-assembling coat with biocompatible liquid lubricant dimethyl silicone oil. The contact angle of gradient’s surface at different temperatures was measured. The transportation behaviors of both water and dimethyl silicone oil on the wettability gradient’s surface were investigated; the results illustrate that the wettability gradient’s slippery surface can successfully self-lubricate from regions with low pillar density to regions with high pillar density, ascribed to the unbalanced Young’s force. The anti-sticking capability of the electrosurgical scalpel with self-lubricating slippery surface was tested. Both the adhesion force and adhesion mass under different cycles were calculated. The results suggest that the as-prepared slippery surface has excellent anti-sticking ability associated with better durability.

## 1. Introduction

Minimally invasive surgery is becoming increasingly popular as it produces less pain, less trauma and rapid recovery for patients. However, soft tissue sticking always occurs on the surface of electrosurgical instruments, such as electrosurgical scalpels, monopole electrode, electrocoagulation and bipolar forceps [1,2,3,4,5]. Owing to the high operating temperature, soft tissue can easily char and adhere to the instrument surface, which may trigger failure of hemostasis or carbonized eschar; leading to unexpected surgical trauma and potential danger to patients [6,7,8,9]. Therefore, it is an urgent need to handle the problems caused by soft tissue sticking during surgical treatment. Several attempts have been made aiming to reduce sticking of soft tissue on electrosurgical scalpels. Based on the current research results, there are two main ways to alleviate tissue sticking: chemical approaches and physical methods. Chemical approaches include adopting low surface energy alloy coating or polymer coating, such as copper-doped diamond-like carbon [10,11,12,13], CrN [14], TiO_2_ [6,15,16,17], poly(l-lactic acid)-polyethylene glycol [18], polygalacturonic acid-1-ethyl-3-(3-dimethylaminopropyl) carbodiimide [19], polylactide–polyethylene glycol tri-block copolymer [20]. Physical methods mainly refer to the design and fabrication of micro/nanostructures on the surface of scalpels [16,21]. However, there are some deficiencies in both approaches such as the limited ability to resist tissue adhesion and the low reliability of alloy coatings that may decompose and release noxious substances under high temperatures. The strength of micro/nanostructures on the surface is insufficient and easily destroyed, failing to work effectively. Hence, it is significant to explore novel methods that promote anti-sticking of soft tissue on electrosurgical scalpels.

Liquid-infused surfaces have a wide range of applications, such as biosensors [22], blood-contacting medical devices [23,24], microfluidic devices [25] and so on [26]. Previous studies have demonstrated a marvelous anti-sticking capacity of the liquid-infused surface for biofouling, ice, frost and varieties of other liquids [27,28,29,30,31,32]. Recently, novel slippery anti-adhesion liquid-infused surfaces which are enhanced by the ultra-slippery property of the peristome of *Nepenthes alata*, have been fabricated by constructing a liquid/solid composite surface. Liquid-infused surfaces require the combination of a micro/nanostructure and the lubricant. The function of a micro/nanostructure is to hold the lubricant firmly, aiming to maintain sufficient and stable lubrication and thus making a liquid/object interface with an anti-sticking effect. Evidence suggests that an adequate and stable lubricant has an important role on the anti-sticking durability of liquid-infused surfaces [33,34,35,36]. Nevertheless, the research above did not fully clarify how to resolve the short term resistance of the lubricant.

**Figure 1:**
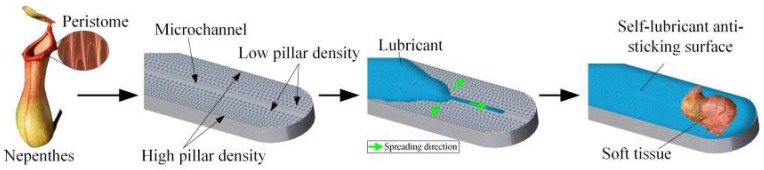
Nepenthes-inspired self-lubricating anti-sticking surface with wettability gradients.

Herein, a liquid-infused slippery surface with wettability gradients is proposed to enhance its anti-sticking function. Non-uniformly distributed cylindrical micro pillars on the surface of electrosurgical scalpels are designed to drive lubricant spreading spontaneously along the microstructure as shown in Figure 1. These wettability gradient microstructures are fabricated via a photolithography-assisted electrolytic etching process, and then a micro channel is generated by a laser process. Silicon oil is chosen as the lubricant for its outstanding biocompatibility and high-temperature resistance [17,37]. A thin layer of octadecyltrichlorosilane (OTS, CH_3_(CH_2_)_16_CH_2_SiCl_3_), which has a few nanometer thickness and strong siloxane bond, is grafted on the wettability gradient’s surface to strongly maintain the lubricant. Behavior of liquid transportation on the as-prepared surface is investigated, and finally the anti-sticking effects of the self-lubricating wettability gradient’s surface are explored by adhesion force measurements and cycle tests.

## 2. Materials and Methods

Electrosurgical scalpels which are made of 316L stainless steel were commercially obtained from Beijing ENJOY Technologies Company Limited (Beijing, China) and were used as the typical electrosurgical instruments in this paper. Positive photoresist (BP212-37S) and its developer were purchased from Kempur Microelectronics Inc. (Beijing, China). Polydimethylsiloxane (PDMS, Dow Corning, Midland, MI, USA) was used as received. NaOH (Sodium hydroxide), Na_2_CO_3_ (Sodium carbonate), H_3_PO_4_ (Phosphoric acid), HCl (Hydrochloric acid), FeCl_3_ (Ferric chloride), *n*-hexane, acetone, ethanol and toluene were supplied by Beijing Chemical Works (Beijing, China). Octadecyltrichlorosilane (OTS) which was used as the silane coupling agent, was purchased from Chengdu Chemical Reagent (Chengdu, China). Dimethyl silicone oil with a viscosity of 100 ± 5 mm^2^/s, provided by Sinopharm Chemical Reagent (Shanghai, China), was used as the infused lubricant. Fresh resected pig liver was chosen as soft tissue materials to facilitate the measurement of adhesion force and tissue adhesion mass, for its relatively homogeneous composition.

**Figure 2:**
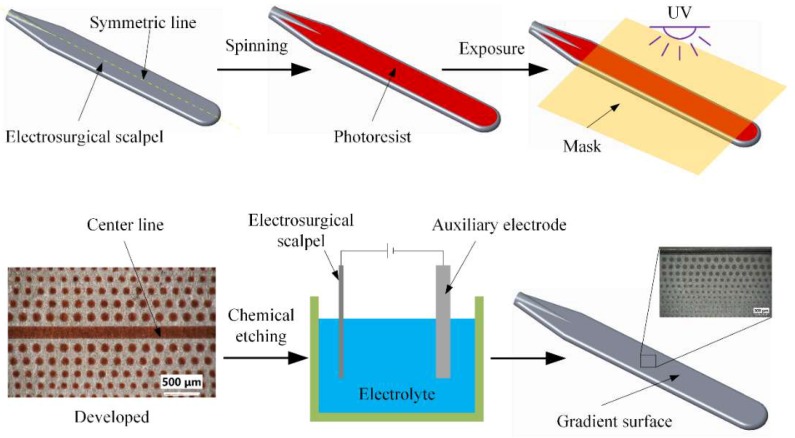
Schematic of non-uniformly distributed cylindrical micro pillars fabrication.

Photolithography-assisted electrolytic etching process was applied to fabricate non-uniformly distributed cylindrical micro pillars on the surface of electrosurgical scalpels. Figure 2 shows the schematic of the manufacturing process. The scalpel was entirely immersed into alkaline solutions with a composition of NaOH 50 g/L and Na_2_CO_3_ 40 g/L for 30 min to first eliminate the oil stain. Then it was thoroughly cleaned by deionized water, *n*-hexane, acetone and ethanol for 10 minutes in proper sequence, respectively. It was subsequently completely dried by vacuum drying at 150 °C for approximately 30 min before spinning. The positive photoresist was spun onto the stainless steel at the speed of 700 rpm for 9 s and 1500 rpm for 15 s to maintain a consistency of thickness, which is about 10 μm. The scalpel was then placed on the hotplate to prebake at the temperature of 120 °C for 3 min. The photolithography process was followed by a contact aligner with ultraviolet (UV) light wavelength of 254 nm and light intensity of 13 mW/cm^2^ for 25 s. In this process, it is necessary to ensure that the central line of the mask and the symmetrical line of the scalpel coincides with each other, to make the wettability gradient’s microstructure distribute perpendicularly to the symmetric line. Postbaking was exerted at 120 °C for 2 min and afterward the scalpel was immersed with photoresist in the stripper for 10 minutes to develop. The electrolytic etching process was applied in an electrolytic cell with about 500 mL electrolyte with a composition of FeCl_3_ 400 g/L, Phosphoric acid 20 g/L and Hydrochloric acid 100 g/L for 30 s under a 0.5 A current. Following the electrolytic etching process the scalpel was removed and rinsed with deionized water. Then the gradient’s microstructures were obtained after removing the photoresist from the scalpel, via ultrasonic cleaning in acetone for approximately 5 min.

**Figure 3:**
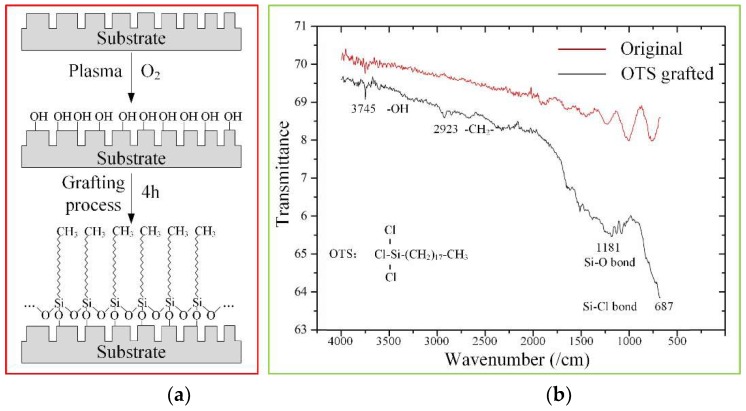
Surface modification of the substrate. (**a**) Schematic of self-assembly thin layer of octadecyltrichlorosilane (OTS) grafting process; (**b**) ATR-FTIR spectra of the substrate before (red line) and after (dark line) self-assembled thin layer of OTS grafts.

Silicon oil was selected as the lubricant for its outstanding biocompatibility and ability to withstand high temperatures. In order to construct a stable slippery wettability gradient surface, silicon oil should be firmly held in the surface. Thus, the property of the substrate must be functionalized to match the silicon oil closely. The silicophilic functionalized property can be realized via grafting a thin, self-assembling layer of OTS. It’s few nanometer thickness, strong bond (siloxane bond) with the substrate [17,37] and the schematic of grafting process, is shown in Figure 3a. Oxygen plasma treatment for 10 min with RF power 100 W, pressure 100 μbar and flow rate 20 sccm after the substrate was thoroughly cleaned, was used to increase the hydroxyl content of the surface; in order to facilitate the formation of OTS self-assembly in the next step. Next the substrate was dipped into anhydrous toluene solution with an OTS concentration of 1 mM/L for 4 h. Then the substrate was taken out and the excess OTS was rinsed using anhydrous toluene. It was then dried under a nitrogen atmosphere. Figure 3b shows the Attenuated Total Reflectance Fourier Transform Infrared (ATR-FTIR) spectra of the substrate before (red line) and after (dark line) the OTS graft. The position and broadening of the Si–O and Si–Cl group vary depending on how the OTS binds at the surface. In the ATR-FTIR spectra of both original substrate and OTS grafted substrate, defined peaks attributed to $\nu_{\mathrm{Si}–O}=1181\;{\mathrm{cm}}^{-1}$ and $\nu_{\mathrm{Si}–\mathrm{Cl}}=687\;{\mathrm{cm}}^{-1}$ were presented. Compared with the spectrum of the original substrate, the grafted surface-bound films suggested a binding mode due to peaks at $\nu_{\mathrm{Si}–O}=1181\;{\mathrm{cm}}^{-1}$ and $\nu_{C–H}=2923\;{\mathrm{cm}}^{-1}$ [38] which demonstrated that the self-assembled thin layer of OTS was successfully grafted onto the substrate.

## 3. Results and Discussion

**Figure 4:**
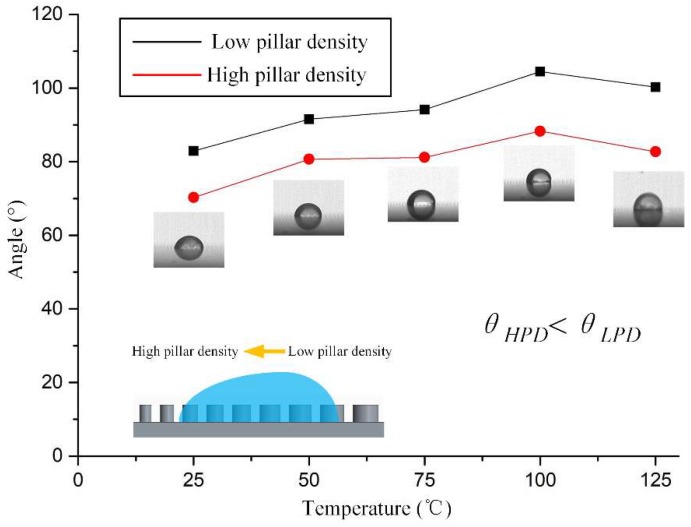
The static contact angle (CA) and asymmetric CA profiles along the wettability gradient’s orientation on the dry surface, under different temperatures conditions.

The wettability gradients can drive droplet transport direction due to the microstructures in the surface [39,40]. The diameters of non-uniformly distributed cylindrical micro pillars range from 70 μm to 150 μm with about 10 μm height. The center-to-center space between pillars ranges from 195 μm in the region with low pillar density, to 125 μm in the region with high pillar density, over a span of 1.5 mm and symmetrically distributed on both sides of the symmetric line. The lubricant was delivered continuously along the microchannel in the center and spread spontaneously on the wettability gradients. These gradients, associated with the functionalized surface, promote an expected outstanding durability of the scalpel’s self-lubricating anti-sticking ability. To explore the mechanisms of droplet spreading behaviors, we analyzed the law of droplet contact angle (CA) by adding a water droplet of 0.5 μL on the dry functionalized gradient’s surface, at temperatures from 25 °C to 125 °C. The gradient substrate was placed on a hotplate before it was placed horizontally. The measurements of CA were taken with the help of a high-speed camera (I-speed LT, Olympus, Tokyo, Japan), which was mounted by a support and adjusted horizontally to match the surface of substrate [41,42]. The morphology of how the droplet initially contacted the surface was recorded. Figure 4 shows the static CA and asymmetric CA profiles along the wettability gradient’s orientation under different temperature conditions. The measured apparent contact angle of regions with high pillar density $\theta_{HPD}$ and low pillar density $\theta_{LPD}$ were 70 ± 0.39° and 82 ± 0.88° at 25 °C, 81 ± 0.3° and 92 ± 0.69° at 50 °C, 81 ± 0.21° and 94 ± 0.71° at 75 °C, 88 ± 0.16° and 104 ± 0.28° at 100 °C, 83 ± 0.64° and 101 ± 0.7°at 125 °C, respectively. According to Figure 4, the water droplet spread exhibited an asymmetric shape and the liquid CA $\theta_{HPD}$ in the region with high pillar density is obviously less than $\theta_{LPD}$ in the region with low pillar density.

The underlying mechanism for droplet spreading was analyzed. Based on the CA, we determined that the droplet was in Wenzel state [43,44]. Under this condition, we can have:

(1) $$\cos\theta =R_{f}\cos\theta_{0}$$

where $R_{f}$ represents the surface roughness, θ and $\theta_{0}$ represent the apparent and intrinsic contact angle of rough and smooth surfaces, respectively. The driving force generated by surface energy gradients owing to the difference of surface roughness can be depicted as [45]:

(2) $$F=\int_{L_{LPD}}^{L_{HPD}}\gamma \left(\cos\theta_{HPD}-\cos\theta_{LPD}\right)dl$$

where *γ* represents the surface tension of the droplet, $\theta_{HPD}$ and $\theta_{LPD}$ represent the advancing and receding angles of the droplet along the wettability gradient’s direction respectively, *dl* represents the integration variable from the region with low pillar density $L_{LPD}$ to the region with high pillar density $L_{HPD}$. As $\theta_{HPD}$ is more miner than $\theta_{LPD}$, we can have the unbalanced Young’ force *F* which, derived from the gradients, will exhibit the drifting trend of the droplet on the surface.

**Figure 5:**
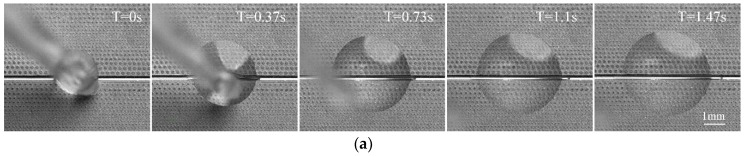

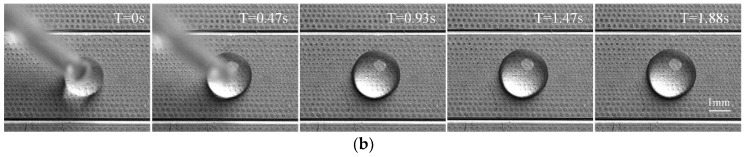
Silicone oil spreading morphology when added to different gradient regions on the functionalized surface: (**a**) Silicone oil spreading behavior when added to the region with low pillar density; (**b**) silicone oil spreading behavior when added to the region with high pillar density.

The unidirectional liquid transport of the wettability gradient’s surface was further investigated. For the silicophilic treated substrate of the scalpel, enough silicone oil was added to the gradient’s surface and ensured that the volume of oil can almost cover the entire gradient’s microstructures. In this procedure, the substrate should be maintained at a horizontal level in order to eliminate any effect of gravity. According to Figure 5a, when oil was added to the region with low pillar density, it could spread spontaneously and rapidly, moving to the region with high pillar density within 1.47 s. While different from Figure 5a, oil stayed stationary when it was added to the region with high pillar density, shown in Figure 5b. This different spreading behavior is consistent with the above theoretical analysis indicating that the wettability gradient’s surface is effective for self-lubrication.

**Figure 6:**
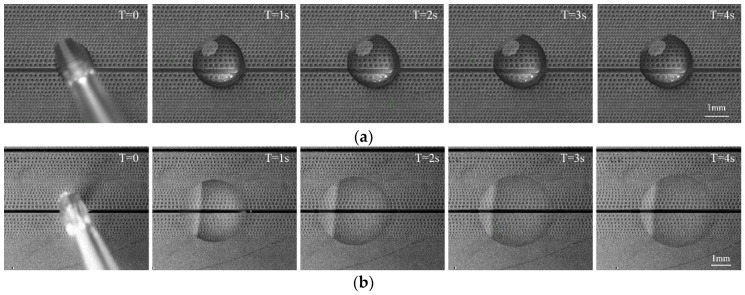
Spreading behavior of silicone oil on unmodified wettability surface and functionalized wettability surface: (**a**) Silicone oil spreading on unmodified wettability surface; (**b**) silicone oil spreading on functionalized wettability surface.

For the functionality of the wettability gradient’s surface, the OTS self-assembled thin layer is critical for the spreading of silicone oil. The grafted Si–O bonds make the surface chemical properties match the silicone oil’s affinitive inducing silicophilic capability, to ensure the substrate is sufficiently lubricated. To examine the effect of an OTS thin layer, we investigated the silicone oil’s spreading behaviors on an unmodified wettability surface and a functionalized wettability surface, as shown in Figure 6. Silicone oil with a volume of 2 μL and viscosity of 100 ± 5 mm^2^/s was added to the unmodified surface via pipette and it stayed originally even in the lowest pillar density region as seen in Figure 6a. Nevertheless, when the silicone oil was added on the OTS self-assembly wettability gradient’s surface, it could stretch completely over the span of the gradient’s distributed micro pillars along the microchannel; presented in Figure 6b. This indicates that the self-lubricating slippery wettability gradient’s surface on the scalpel was successfully fabricated.

**Figure 7:**
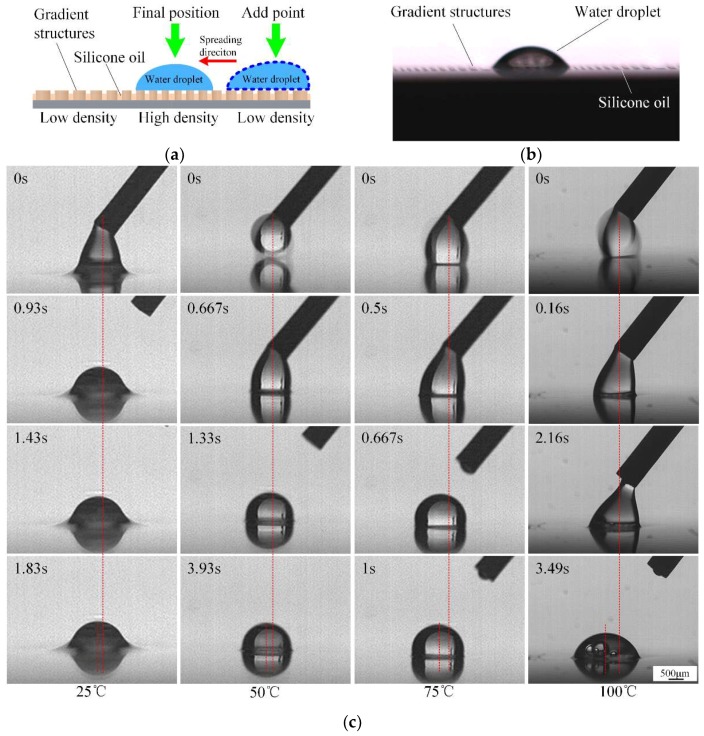
Motion behavior of a droplet on the slippery wettability gradient’s surface: (**a**) Diagram of initial and steady position of the added liquid; (**b**) optical photograph of liquid morphology on the slippery wettability gradient’s surface; (**c**) water spreading tests at different temperatures.

The amount of silicon oil stored on the surface may be consumed gradually during contact between the soft tissue and the gradient substrate. Consequently, a continuous supply of lubricant on the gradient’s surface is crucial to sustain the anti-sticking capacity of the as-prepared surface. In order to vividly express continuous supply of lubricant on the gradient’s surface, and make a distinct observation distinguished from the residue silicone oil, a water droplet was chosen as the alternative lubricant. Results show that when adding a water droplet to the oil-infused gradient’s surface, it can easily slide; propelled by the unbalanced Young’s force. Figure 7a illustrates the motion behavior of the droplet moving directionally on the slippery wettability gradient’s surface and its optical photograph is shown in Figure 7b. For the slippery wettability gradient’s surface, the water droplet gradually slid from the region with low pillar density to the region with high pillar density and ultimately remained stationary. The red-dashed lines labeled in Figure 7c represent the deviation distances center-to-center of the droplet. The deviation distance of the droplet was about 188 ± 5 μm within 1.43 s at a temperature of 25 °C and 230 ± 2.45 μm within 3.93 s, 326 ± 4.88 μm within 1 s, 452 ± 2.86 μm within 3.49 s at temperature of 50 °C, 75 °C, and 100 °C, respectively. The deviation values are shown in Table 1 and each came from three individual measurements.

**Figure 8:**
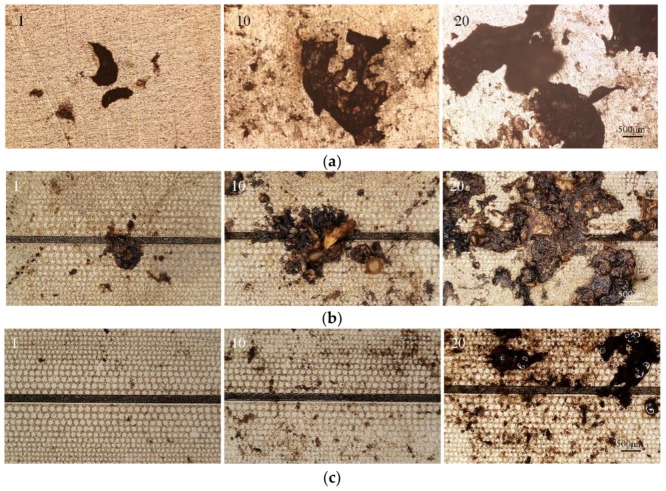
Soft tissue cutting cycle tests: (**a**) Tissue sticking on smooth surface at the first, 10th, 20th cycle tests; (**b**) tissue sticking on dry gradient surface at the first, 10th, and 20th cycle tests; (**c**) tissue sticking on self-lubricating slippery wettability gradient’s surface at the first, 10th, and 20th cycle tests.

Experiments of tissue cutting cycle tests were carried out to evaluate the anti-sticking effects of the as-prepared surface. In order to facilitate the operating process and precisely test the adhesion force and adhesion mass, fresh resected pig liver was cut into blocks with size of 3 cm × 3 cm, approximately 1 cm thickness and fixed on a digital force gauge (HANDPI-50, AIDEBAO, China) which was mounted on a mobile base controlled by a micromanipulator (MX7600R, SISKIYOU, Grants Pass, OR, USA). The test samples were fixed on a temperature-controlled hotplate. Before the tissue cutting test, the test sample was heated to 300 degrees [7] and held for five minutes to equalize the temperature. The weight of the test samples was measured with a high resolution electric scale before and after the tests to evaluate the adhesion mass. For the first test, the soft tissue was loaded on the test surface with 5 N and then unloaded at a speed of 500 μm/s. The unloading pressures were recorded and seen as the adhesion forces. Each adhesion force and adhesion mass came from three individual measurements. Figure 8a shows the tissue sticking for the first time, during the 10th and 20th cycle test on the control smooth surface, respectively. Figure 8b shows the tissue sticking for the first time, during the 10th and 20th cycle test on the dry gradient’s surface, respectively. Due to direct contact with the dry substrate, soft tissue can easily stick for the first time and the sticking area will enlarge rapidly as experiments go on. As seen in Figure 8a,b, at the 20th cycle test, both kinds of surfaces were seriously stuck and greatly affected cutting functions. Tissue sticking on self-lubricating slippery wettability gradient’s surface is shown in Figure 8c. Ascribed to the anti-sticking capability of the substrate, soft tissue rarely adhered to the surface for the first test and slightly stuck in the 10th cycle test. The lubricant stuck on the surface affected due to the anti-sticking durability of the surface. Microchannels on the self-lubricating slippery wettability gradient’s surface can maintain the plenitudinous supply of lubricant and ensure better durability. Compared to the dry surface, the soft tissue sticking on the self-lubricating wettability surface was alleviated in the 20th cycle test, in accordance with Figure 8c.

**Figure 9:**
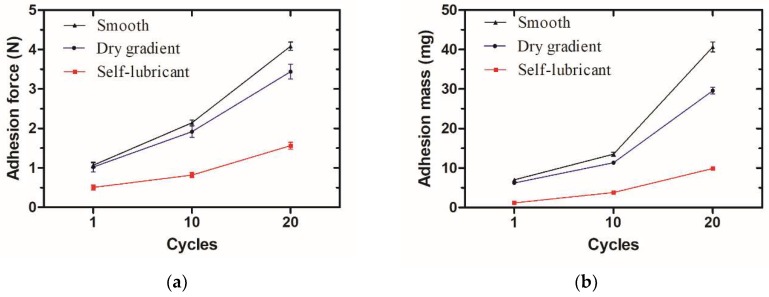
Contrast of anti-sticking stability between two kinds of surfaces: (**a**) Graph of adhesion force variance on the smooth, dry gradient and self-lubricating surface in cycle tests; (**b**) graph of adhesion mass accumulation of the smooth, dry gradient and self-lubricating surface in cycle tests.

The adhesion force and the adhesion mass were measured in the cycle tests. Based on Figure 9a, the adhesion force of the self-lubricating slippery wettability gradient’s surface is about 0.51 ± 0.07 N and significantly reduced by approximately 90% compared with the smooth and dry gradient surface. As the cycles increases, adhesion force on the smooth and dry gradient surfaces drastically increase and the smooth surface shows a larger adhesion force than the dry gradient surface, while the self-lubricating surface possessed relatively low adhesion force for the stable lubricant supply capability. Figure 9b shows the adhesion mass of the cycle tests. The self-lubricating surface exhibited minute adhesion mass in contrast to smooth and dry gradient surfaces for the first cycle test, due to the excellent anti-sticking properties. The adhesion mass on the self-lubricating surface at 20th cycle was 9.9 ± 0.26 mg, which was far less than that 40.7 ± 1.26 mg on the smooth surface and 29.6 ± 0.85 mg on the dry gradient’s surface, which suggests a long durability for soft tissue sticking.

## 4. Conclusions

In summary, we designed and fabricated surfaces with wettability gradients on the substrate of electrosurgical scalpels with a photolithography-assisted electrolytic etching process, which consisted of non-uniform cylindrical micro pillars distributed on both sides of a symmetric line. An OTS self-assembling thin layer was successfully grafted on the substrate and played an important role in silicone oil spreading on the functionalized surface. Experiments were conducted to explore the motion behavior of the wettability gradient’s surface and we found that the liquid stretched from the region with low pillar density to the region with high pillar density; indicating that the self-lubricating surface was successfully prepared. The underlying mechanisms of water self-propulsion were further analyzed. Experiments of fresh resected pig liver cutting were carried out to test the anti-sticking ability of the as-prepared surface. Results revealed that the scalpel with a self-lubricating surface expressed a marvelous anti-sticking performance, the adhesion force was significantly reduced and the durability prolonged. We believe that this approach will provide new insight into constructing anti-sticking surfaces including industry, microfluidic system, biochips or other practical applications.

## Figures and Tables

**Table 1:** Deviation distance of droplet along the gradient’s orientation.

**Temperature (°C)**	25	50	75	100
**Deviation (μm)**	188 ± 5	230 ± 2.45	326 ± 4.88	452 ± 2.86

## References

[1] Massarweh N.N., Cosgriff N., Slakey D.P. (2006). Electrosurgery: History, principles, and current and future uses. J. Am. Coll. Surg..

[2] Zhang P., Liu G., Zhang D., Chen H. (2018). Liquid-infused surfaces on electrosurgical instruments with exceptional antiadhesion and low-damage performances. ACS Appl. Mater. Interfaces.

[3] Scott J.E., Swanson E.A., Cooley J., Wills R.W., Pearce E.C. (2017). Healing of canine skin incisions made with monopolar electrosurgery versus scalpel blade. Vet. Surg..

[4] Choi M.Y., Koo I.G., Kim P.Y., Kang S.K., Kim Y.-S., Jung J.-C., Collins G.J. (2012). Helium/H_2_O_2_ atmospheric pressure plasma-assisted electrosurgery. Plasma Process. Polym..

[5] Kang S.K., Kim P.Y., Koo I.G., Kim H.Y., Jung J.-C., Choi M.Y., Lee J.K., Collins G.J. (2012). Non-stick polymer coatings for energy-based surgical devices employed in vessel sealing. Plasma Process. Polym..

[6] Han Z., Fu J., Feng X., Niu S., Zhang J., Ren L. (2017). Bionic anti-adhesive electrode coupled with maize leaf microstructures and TiO_2_ coating. RSC Adv..

[7] Zhang P., Chen H., Zhang L., Zhang D. (2016). Anti-adhesion effects of liquid-infused textured surfaces on high-temperature stainless steel for soft tissue. Appl. Surf. Sci..

[8] Sutton P.A., Awad S., Perkins A.C., Lobo D.N. (2010). Comparison of lateral thermal spread using monopolar and bipolar diathermy, the harmonic scalpel and the ligasure. Br. J. Surg..

[9] Sotiri I., Overton J.C., Waterhouse A., Howell C. (2016). Immobilized liquid layers: A new approach to anti-adhesion surfaces for medical applications. Exp. Biol. Med..

[10] Han Yi Cheng K.L.O. (2015). The application of advanced nanostructured film in electrosurgical device: Anti-sticking behavior and thermal injury. J. Nanomed. Nanotechnol..

[11] Hsiao W.T., Lin L.H., Chiang H.J., Ou K.L., Cheng H.Y. (2015). Biomedical electrosurgery devices containing nanostructure for minimally invasive surgery: Reduction of thermal injury and acceleration of wound healing for liver cancer. J. Mater. Sci. Mater. Med..

[12] Lin L.-H., Hsu Y.-J., Chiang H.-J., Cheng H.-Y., Wang C.-S., Ou K.-L. (2015). The application of minimally invasive devices with nanostructured surface functionalization: Antisticking behavior on devices and liver tissue interface in rat. J. Nanomater..

[13] Shen Y.D., Lin L.H., Chiang H.J., Ou K.L., Cheng H.Y. (2016). Research of electrosurgical unit with novel antiadhesion composite thin film for tumor ablation: Microstructural characteristics, thermal conduction properties, and biological behaviors. J. Biomed. Mater. Res. B Appl. Biomater..

[14] Ou K.L., Chu J.S., Hosseinkhani H., Chiou J.F., Yu C.H. (2014). Biomedical nanostructured coating for minimally invasive surgery devices applications: Characterization, cell cytotoxicity evaluation and an animal study in rat. Surg. Endosc..

[15] Wooh S., Butt H.J. (2017). A photocatalytically active lubricant-impregnated surface. Angew. Chem. Int. Ed. Engl..

[16] Han Z., Fu J., Fang Y., Zhang J., Niu S., Ren L. (2017). Anti-adhesive property of maize leaf surface related with temperature and humidity. J. Bionic. Eng..

[17] Phan N., Moronuki N. (2017). Fabrication of high aspect ratio silicon micro-/nano-pore arrays and surface modification aiming at long lifetime liquid-infused-type self-cleaning function. J. Adv. Mech. Des. Syst. Manuf..

[18] Lee J.H., Go A.K., Oh S.H., Lee K.E., Yuk S.H. (2005). Tissue anti-adhesion potential of ibuprofen-loaded PLLA-PEG diblock copolymer films. Biomaterials.

[19] Lee M.W., Hung C.L., Cheng J.C., Wang Y.J. (2005). A new anti-adhesion film synthesized from polygalacturonic acid with 1-ethyl-3-(3-dimethylaminopropyl)carbodiimide crosslinker. Biomaterials.

[20] Yang D.J., Chen F., Xiong Z.C., Xiong C.D., Wang Y.Z. (2009). Tissue anti-adhesion potential of biodegradable PELA electrospun membranes. Acta Biomater..

[21] Lin C.C., Lin H.J., Lin Y.H., Sugiatno E., Ruslin M., Su C.Y., Ou K.L., Cheng H.Y. (2017). Micro/nanostructured surface modification using femtosecond laser pulses on minimally invasive electrosurgical devices. J. Biomed. Mater. Res. B Appl. Biomater..

[22] Hosseini A., Villegas M., Yang J., Badv M., Weitz J.I., Soleymani L., Didar T.F. (2018). Conductive electrochemically active lubricant-infused nanostructured surfaces attenuate coagulation and enable friction-less droplet manipulation. Adv. Mater. Interfaces.

[23] Badv M., Jaffer I.H., Weitz J.I., Didar T.F. (2017). An omniphobic lubricant-infused coating produced by chemical vapor deposition of hydrophobic organosilanes attenuates clotting on catheter surfaces. Sci. Rep..

[24] Badv M., Imani S.M., Weitz J.I., Didar T.F. (2018). Lubricant-infused surfaces with built-in functional biomolecules exhibit simultaneous repellency and tunable cell adhesion. ACS Nano.

[25] Villegas M., Cetinic Z., Shakeri A., Didar T.F. (2018). Fabricating smooth pdms microfluidic channels from low-resolution 3D printed molds using an omniphobic lubricant-infused coating. Anal. Chim. Acta.

[26] Zhao Y., Wang H., Zhou H., Lin T. (2017). Directional fluid transport in thin porous materials and its functional applications. Small.

[27] Yong J., Chen F., Yang Q., Huo J., Hou X. (2017). Superoleophobic surfaces. Chem. Soc. Rev..

[28] Xu Q., Zhang W., Dong C., Sreeprasad T.S., Xia Z. (2016). Biomimetic self-cleaning surfaces: Synthesis, mechanism and applications. J. R. Soc. Interface.

[29] Zheng Y., Bai H., Huang Z., Tian X., Nie F.Q., Zhao Y., Zhai J., Jiang L. (2010). Directional water collection on wetted spider silk. Nature.

[30] Ju J., Bai H., Zheng Y., Zhao T., Fang R., Jiang L. (2012). A multi-structural and multi-functional integrated fog collection system in cactus. Nat. Commun..

[31] Muschi M., Brudieu B., Teisseire J., Sauret A. (2018). Drop impact dynamics on slippery liquid-infused porous surfaces: Influence of oil thickness. Soft Matter.

[32] Togasawa R., Tenjimbayashi M., Matsubayashi T., Moriya T., Manabe K., Shiratori S. (2018). A fluorine-free slippery surface with hot water repellency and improved stability against boiling. ACS Appl. Mater. Interfaces.

[33] Wong T.S., Kang S.H., Tang S.K., Smythe E.J., Hatton B.D., Grinthal A., Aizenberg J. (2011). Bioinspired self-repairing slippery surfaces with pressure-stable omniphobicity. Nature.

[34] Chen H., Zhang P., Zhang L., Liu H., Jiang Y., Zhang D., Han Z., Jiang L. (2016). Continuous directional water transport on the peristome surface of Nepenthes alata. Nature.

[35] Zhang P., Zhang L., Chen H., Dong Z., Zhang D. (2017). Surfaces inspired by the nepenthes peristome for unidirectional liquid transport. Adv. Mater..

[36] Juuti P., Haapanen J., Stenroos C., Niemelä-Anttonen H., Harra J., Koivuluoto H., Teisala H., Lahti J., Tuominen M., Kuusipalo J. (2017). Achieving a slippery, liquid-infused porous surface with anti-icing properties by direct deposition of flame synthesized aerosol nanoparticles on a thermally fragile substrate. Appl. Phys. Lett..

[37] Zhang P., Chen H., Zhang L., Zhang Y., Zhang D., Jiang L. (2016). Stable slippery liquid-infused anti-wetting surface at high temperatures. J. Mater. Chem. A.

[38] Heib F., Hempelmann R., Munief W.M., Ingebrandt S., Fug F., Possart W., Groß K., Schmitt M. (2015). High-precision drop shape analysis (HPDSA) of quasistatic contact angles on silanized silicon wafers with different surface topographies during inclining-plate measurements: Influence of the surface roughness on the contact line dynamics. Appl. Surf. Sci..

[39] Liu C., Sun J., Li J., Xiang C., Che L., Wang Z., Zhou X. (2017). Long-range spontaneous droplet self-propulsion on wettability gradient surfaces. Sci. Rep..

[40] Zhou K., Zhu X.G., Li Y., Liu J. (2014). Fabrication of PDMS micro through-holes using micromolding in open capillaries. RSC Adv..

[41] Schulze R.D., Possart W., Kamusewitz H., Bischof C. (1989). Young’s equilibrium contact angle on rough solid surfaces. Part I. An empirical determination. J. Adhes. Sci. Technol..

[42] Koch B.M., Elliott J.A., Amirfazli A. (2014). Study of model superoleophobic surfaces fabricated with a modified bosch etch method. Langmuir.

[43] WENZEL R.N. (1936). Resistance of solid surfaces to wetting by water. Ind. Eng. Chem..

[44] Wu J., Ma R., Wang Z., Yao S. (2011). Do droplets always move following the wettability gradient?. Appl. Phys. Lett..

[45] Chaudhury M.W., Whitesides G.M. (1992). How to make water uphill. Science.

